# Characterization of Aldehyde Oxidase (AO) Genes Involved in the Accumulation of Carotenoid Pigments in Wheat Grain

**DOI:** 10.3389/fpls.2017.00863

**Published:** 2017-05-24

**Authors:** Pasqualina Colasuonno, Ilaria Marcotuli, Maria L. Lozito, Rosanna Simeone, Antonio Blanco, Agata Gadaleta

**Affiliations:** ^1^Department of Agricultural and Environmental Science, University of Bari Aldo MoroBari, Italy; ^2^Department of Soil, Plant and Food Sciences, University of Bari Aldo MoroBari, Italy

**Keywords:** wheat, carotenoid genes, aldehyde oxidase, SNP, yellow pigments

## Abstract

Aldehyde Oxidase (AO) enzyme (EC 1.2.3.1) catalyzes the final steps of carotenoid catabolism and it is a key enzyme in the abscisic acid (ABA) biosynthesis. AO isoforms are located in the cytosolic compartment of tissues in many plants, where induce the oxidation of aldehydes into carboxylic acid, and in addition, catalyze the hydroxylation of some heterocycles. The goal of the present study was to characterize the *AO* genes involved in the accumulation of carotenoid pigments in wheat grain, an important quantitative trait controlled by multiple genes. The cDNAs corresponding to the four *AO* isoforms from *Arabidopsis thaliana* and five *AO* isoforms from *Brachypodium distachyon* were used as query in 454 sequence assemblies data for *Triticum aestivum* cv. Chinese Spring (https://urgi.versailles.inra.fr/blast/blast.php) to obtain the partial or whole orthologous wheat *AO* sequences. Three wheat isoforms, designated *AO1, AO2*, and *AO3* were located on the chromosome groups 2, 5, and 7, respectively, and mapped on two consensus wheat maps by SNP markers located within the *AO* gene sequences. To validate the possible relationships between *AO3* genes and carotenoid accumulation in wheat, the expression levels of *AO-A3* and *AO-B3* gene were determined during the kernel maturation stage of two durum wheat cultivars, Ciccio and Svevo, characterized by a low and high carotenoid content, respectively. Different *AO-A3* gene expression values were observed between the two cultivars indicating that the *AO-A3* allele present in Ciccio was more active in carotenoid degradation. A gene marker was developed and can be used for marker-assisted selection in wheat breeding programs.

## Introduction

Yellow pigment concentration (YPC) in wheat is a quantitative trait controlled by a complex genetic system and influenced by environmental factors (Qin et al., 2016). The yellow color of grain and flour is mainly due to carotenoid accumulation in the pericarp and endosperm. Carotenoids are precursors of the vitamin A, with high nutritional relevance for human diet (Della Penna and Pogson, 2006; Britton, 2009), and substrates for the synthesis of apocarotenoids, compounds derived from oxidative cleavage and further modifications (Wurtzel et al., 2012). Apocarotenoids include retinol (vitamin A) and the hormones abscisic acid (ABA) and strigolactones (Rosati et al., 2010).

The carotenoid biosynthesis has been almost completely clarified in *Arabidopsis thaliana*, rice, maize and in some ornamental plants (Hirschberg, 2001; Chaudhary et al., 2010; Ruiz-Sola and Rodriguez-Concepcion, 2012; da Silva Messias et al., 2014; Moise et al., 2014). Briefly, the first committed step of biosynthesis is mediated by the phytoene synthase (PSY) enzyme for the condensation of two molecules of geranylgeranyl diphosphate to produce phytoene. Then, the phytoene goes through a series of desaturation reactions by phytoene desaturase (PDS), zeta-carotene isomerase (Z-ISO), zeta-carotene desaturase (ZDS), and carotenoid isomerase (CRTISO) enzymes, producing lycopene molecules. Double lycopene cyclization can produce precursor of lutein (branch β-ε) by lycopene ε-cyclase (LYCE) or β-carotene (branch β-β) by lycopene ß-cyclase (LYCB) (Qin et al., 2016).

Several QTL located on almost all wheat chromosomes have been detected by linkage mapping using biparental populations, genome wide association study (GWAS) and “candidate genes” approaches using germplasm collections (see review by Colasuonno et al., 2017). Numerous studies have attested the central role of some key genes in the carotenoid pathway and their association to QTL for YPC, such as the (*CRTISO*) gene on chromosomes 1A (Cazzonelli and Pogson, 2010), violaxanthin de-epoxidase (*VDE*) on 2B (Tsilo et al., 2011; Roncallo et al., 2012), lycopene epsilon-cyclase (*LYCE*) on chromosome group 3 (Howitt et al., 2009; Crawford and Francki, 2013), phytoene synthase (*PSY1* and *PSY2*) on chromosomes 7A and 5A (Pozniak et al., 2007; Li et al., 2009).

**Figure 1:**
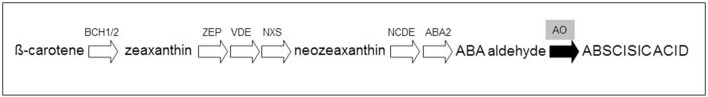
**Simplified representation of the abscisic acid pathway**. The conversion of β-carotene to abscisic acid is catalyzed by the β-carotene hydroxylases (BCH1/2), zeaxanthin epoxidase (ZEP), violaxanthin de-epoxidase (VDE), neoxanthin synthase (NXS), 9-cis-epoxycarotenoid dioxygenase (NCDE) and short-chain alcohol dehydrogenase (ABA2). The last step involves the aldehyde oxidese (AO), responsible of the synthesis of abscisic acid (black arrow).

Oxidative cleavage enzymes involved in carotenoid degradation, sequestration, and storage can also influence the accumulation of carotenoid pigments in tissues (Gonzalez et al., 2013). β-carotene and some of its derivatives can be modified by carotenoid cleavage dioxygenases (CCDs) resulting in the production of strigolactones (Alder et al., 2012). Moreover, a family of carotenoid 9-cis-epoxycarotenoid dioxygenases (NCEDs), which catalyzes the cleavage of carotenoids at specific double bonds, was shown to be active in the production of apocarotenoids (Auldridge et al., 2006). In particular, the ABA, derived from the conversion of violaxanthin by aldehyde oxidase (AO), is involved in regulating plant responses to various environmental stresses (e.g., drought and salinity stress) and long-distance signaling within the plants (Davies et al., 2005; Figure 1).

A major QTL for YPC, accounting for up to 60% of the phenotypic variation, was detected on the long arm of chromosome 7A in different experiments carried out on diversified genetic materials using biparental populations (He et al., 2008, 2009; Zhang and Dubcovsky, 2008). Subsequently, two different QTL were identified on 7AL (Zhang and Dubcovsky, 2008; Blanco et al., 2011; Crawford et al., 2011): the first one on the distal bin 7AL18-0.90-1.00 associated with allelic variation at *Psy1* (He et al., 2008, 2009; Zhang et al., 2009), and the second one on the bin 7AL21-0.74-0.86 linked with allelic variations at *AO* (Colasuonno et al., 2014, 2017).

The members of the AO enzyme (EC 1.2.3.1) play a role into last steps of carotenoid catabolism by oxidation of abscisic aldehyde in (ABA) (Seo et al., 2000a; Figure 1). Indeed, AO enzyme exists in multiple forms expressed in leaves, roots and seeds, depending on the species (Seo et al., 1998, 2000a,b; Xiong et al., 2001). AO proteins have been physiologically studied in *Arabidopsis thaliana* (Seo et al., 2000b), tomato (Min et al., 2000), barley (Omarov et al., 2003), maize (Qin et al., 2013), and partially in common wheat (Gallè et al., 2013).

The objectives of the present study were to: (a) identify the *AO* gene family in wheat and map each member on two wheat high-density consensus maps; (b) characterize *AO3* transcription level by qRT-PCR in kernel tissue of two durum wheat cultivars differing for carotenoid content; (c) develop *AO3* markers suitable for MAS to be used in breeding programs.

## Materials and methods

### Plant materials

A recombinant inbred line (RIL) mapping population, previously developed from the cross Svevo × Ciccio and genotyped using the SNP iSelect array (Colasuonno et al., 2014), was evaluated for the abscisic aldehyde oxidase 3 (AO-A3) gene and yellow pigment content. The elite durum wheat cultivars Svevo and Ciccio were different for qualitative and quantitative traits, such as grain yield components, grain protein content, grain yellow pigments and adaptive traits. Regression analysis, carried out in the Svevo x Ciccio RIL population grown in four environments (Valenzano 2006, Foggia 2006, Valenzano 2007, and Foggia 2007) and implemented in QGene, was used to underlined the association between the abscisic aldehyde oxydase 3 (AO-A3) gene and yellow pigment content. The marker-trait association was considered significant when –log10(P) ≥ 3.0. Phenotypic variation explained by the gene marker (R^2^) and additive effects were investigated. Positive and negative signs on the effect values indicated the contribution of Svevo and Ciccio, respectively, toward higher trait value.

### Aldehyde oxidase (AO) isolation and characterization

The sequences of *AO* genes from *Arabidopsis thaliana* and *Brachypodiun distachyon* were downloaded from the TAIR database (http://arabidopsis.org/) and Phytozome (https://phytozome.jgi.doe.gov/pz/portal.html#!info?alias=Org_Bdistachyon), respectively, and used as query in 454 sequence assemblies data for *Triticum aestivum* cv. Chinese Spring (https://urgi.versailles.inra.fr/blast/blast.php) to obtain partial or whole orthologous wheat *AO* sequences, including the promoter region.

The three wheat AO sequences included in the contigs 2AL_6408908, 5BL_10864426 and 7AL_4455104 were blasted against the available dataset of SNP marker sequences reported by Wang et al. (2014), and SNPs with ≥ 80% identity were considered within the *AO* genes. The high-density linkage maps described by Maccaferri et al. (2015) for durum wheat and by Wang et al. (2014) for common wheat were used as reference maps for determining a detailed map position of *AO* genes.

Wheat AO gene and promoter structure prediction was conducted with the FGENESH program (http://www.softberry.com/berry.phtml?topic=fgenesh&group=programs&subgroup=gfind). Each predicted protein was considered into the next step of analysis.

Further BLASTn analysis for three representative isoforms was extended to Wheat 61k GeneChip in PLEXdb database (http://www.plantgdb.org) for obtaining information on each transcription pattern variation during different wheat developmental stages (Experiment TA3).

The bioinformatics analysis regarding the promoter region was conducted with PlantCARE database (http://bioinformatics.psb.ugent.be/webtools/plantcare/html/) to reveal cis-acting regulatory elements and Plant MITE database (http://pmite.hzau.edu.cn/MITEblast/blast.html) to identify transposon regions.

### Quantitative real-time PCR

In order to pick primer combinations to be used for *AO3* gene expression analysis, cDNA sequences of *AO-A3* (included in the contig 7AL-4455104) and *AO-B3* (accession number Ta_AK331622 from NCBI database) were aligned to highlight dissimilarities between the two homoeologous genes. Specific primer pairs for each gene were designed in a region of the second exon since a number of polymorphisms were detected between the A and B genomes (Table S1).

The genetic material used for the *AO* expression analysis was represented by the durum wheat cultivars Ciccio and Svevo characterized by low and high values of YPC, respectively. Kernel tissues from each cultivar were harvested, frozen in liquid nitrogen and stored at −80°C until RNA extraction. Total RNA was extracted with RNeasy Plant Mini Kit (QIAGEN®) and checked on 1.5% denaturing agarose gel. All RNA samples were lead to the same concentration (1 μg) for subsequent treatment with DNase I recombinant (Roche Applied Science, Mannheim, Germany), in order to remove genomic DNA, and then were reverse-transcribed into double stranded cDNA with Trascriptor First Strand cDNA Synthesis Kit (Roche Applied Science, Mannheim, Germany). Data were normalized using three reference genes: Cell Division Control AAA-Superfamily of ATPases (*CDC*), ADP-Ribosilation Factor (*ADP-RF*), and RNase L Inhibitor-like protein (*RLI*) (Paolacci et al., 2009; Giménez et al., 2011). These genes have a stability value around 0.035 when evaluated with NormFinder software (Andersen et al., 2004).

Quantitative Real-Time PCR analyses were carried out using EVA® GREEN in the CFX96TM Real time PCR Systems (BIO-RAD). The PCR cycle was: 95°C for 3 min followed by 40 cycles of 95°C for 10 s and at 60°C for 30 s. Amplification efficiency (98% to 100%) for the primer set was determined by amplification of cDNA with a series of six scalar dilution (1:5) per reaction. Each 10 μl PCR reaction contained 1 μl of a 1:5 dilution cDNA, 5 μl of EvaGreen Mix 10X (Bio-Rad), and 500 nM of each primer. All experiments were performed in Hard-Shell 96-well skirted PCR plates (HSP9601) with Microseal R “B” Adhesive Seals (MSB-1001) from Bio-Rad. Fluorescence signals were collected at each polymerization step. The specificity of the amplicons was confirmed by the presence of a single band of expected size for each primer pair in agarose gel (2% w/v), by a single peak melting curves of the PCR products and by sequencing of the amplified fragments (3,500 Genetic Analyzer, Applied Biosystems). qRT-PCR data for both genes were derived from the mean values of three independent amplification reactions carried out on five different plants harvested in the same phenotypic stage (biological replicates). All calculations and analyses were performed using CFX Manager 2.1 software (Bio-Rad Laboratories) using the ΔC_*t*_ method, which used the relative quantity (RQ) calculated with a ratio of the RQ of the target gene to the relative expression of the reference gene (including the three reference targets in each sample). Standard deviations were used to normalize values for the highest or lowest individual expression levels (CFX Manager 2.1 software user manual, Bio-Rad Laboratories). The Student's *t*-test was used to underline significant differences between control and treated samples for the two considered *AO3* genes.

### Development of AO3 gene markers for MAS

Primers were designed using Primer3 (http://frodo.wi.mit.edu/primer3/) software on the basis of the 7AL_4455104 contig sequence including the SNP marker IWB59875 previously resulted associated to carotenoid content (Colasuonno et al., 2014). The primer sequences PG42-for (TCTACACACCACGAGACCTT) and PG48-rev (GCAACTCAGCGATCCAACAATAT) amplified a 635 bp fragment.

PCR products derived from the durum cvs. Ciccio and Svevo were loaded on agarose gel to confirm amplicon length, and injected onto the WAVE® system (Transgenomic, Omaha, NE, USA) to undergo check peak intensity of the amplicons. The temperature required for successful resolution of heteroduplex molecules was determined using the DHPLC Navigator™ Software (Transgenomic, Omaha, NE, USA), which considered the specific amplicon sequence and size to calculate the denaturation curve. PCR product was cleaned using ExoSAP-IT (USB, Cleveland, OH, USA) according to manufacturer's instructions, then sequenced using BigDye terminator sequencing kit on a ABI-3500 Genetic Analyzer (Applied Biosystems, Foster City, CA, USA). The detection of molecular marker was performed on DHPLC device in “mutation detection” under “Rapid DNA” run mode.

## Results

### Isolation of genomic sequences of AO genes in wheat

**Figure 2:**
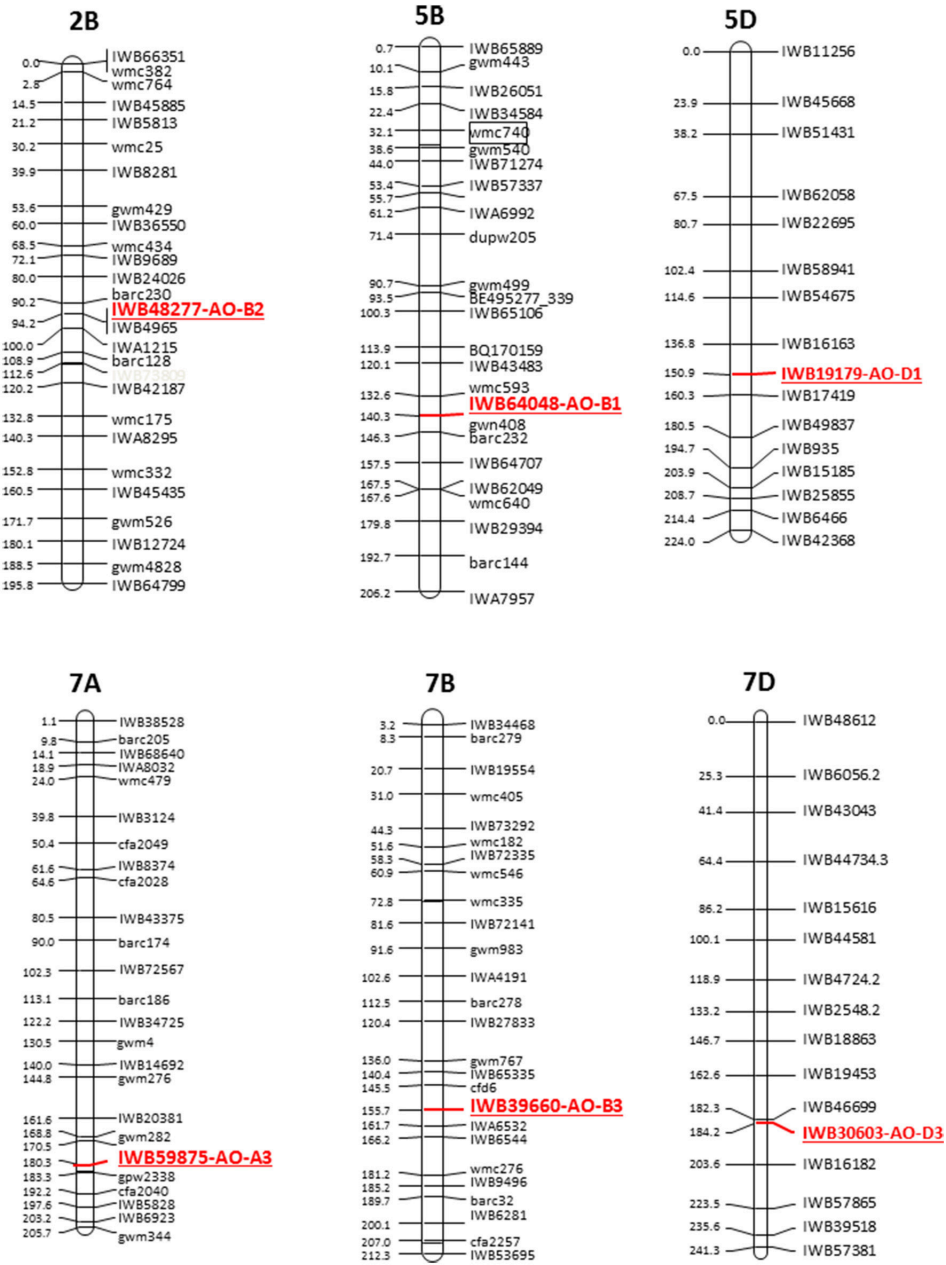
**Schematic representation of the durum wheat linkage map and AO markers**. Each linkage map derives from the durum consensus map (Maccaferri et al., 2015) and has been represented by a SNP marker every about 20 cM. SSR markers have been also inserted every about 20 cM to compare the consensus SNP map with published SSR-based maps.

The cDNAs corresponding to the four *AO* isoforms from *A. thaliana* (AT5G20960, AT3G43600, AT2G27150, and AT1G04580, designated, respectively *O1, AO2, AO3*, and *AO4*) and the five *AO* isoforms from *B. distachyon* (XM010230033, XM_003557870, XM_003559293, and XM_003559295, all designated AO2, and XM_003561213 (designated AO) were used as query in 454 sequence assemblies data for *T. aestivum* cv. Chinese Spring (https://urgi.versailles.inra.fr/blast/blast.php) to obtain the partial or whole orthologous wheat *AO* sequences. The search into the Chinese Spring sequence database released several AO sequence fragments included in 13 different contigs located on the wheat chromosome groups 2, 5, and 7 (Table 1). The definitive assignment of *AO* sequences to the wheat A, B and D homoeologous chromosomes and the accurate map position was performed based on the best blastn hit (percentage identity) with the available dataset of SNP marker sequences reported by Wang et al. (2014) (Table 1). The Recommended Rules for Gene Symbolization reported in the Wheat Catalog (McIntosh et al., 2005) were used for AO nomenclature. In particular, six SNPs corresponding to *AO1* genes mapped on chromosome group 5 (*AO-B1* and *AO-D1*), 12 SNPs within *AO2* mapped on chromosome group 2 (*AO-A2, AO-B2*, and *AO-D2*), and 16 SNP markers mapped on chromosome group 7 (*AO-A3, AO-B3*, and *AO-D3*). Out of 34 SNPs corresponding to *AO* gene sequences, five and nine markers were located on the consensus durum (Maccaferri et al., 2015) and bread wheat maps (Wang et al., 2014), respectively (Figure 2).

**Table 1:** **List of aldehyde oxidase genes in wheat with corresponding contig number, SNP markers, chromosome localization and map position on the durum (Maccaferri et al., **2015**) and bread wheat (Wang et al., **2014**) consensus maps**.

**Gene**	**Carotenoid enzyme**	**SNP name**	**SNP id**	**Allele**	**Contig**	**Wheat map position**		
						**Chrom**	**Durum map**	**Bread map**
*AO-1*	Aldehyde oxidase 1	CAP8_c2820_207	IWB14642	A/G	5BL_10921744	5BL	-	-
		RFL_Contig2772_3038	IWB64051	T/C	5BL_10864426	5BL	-	-
		RFL_Contig2772_2171	IWB64049	A/G	5BL_10864426	5BL	-	-
		RFL_Contig2772_1693	IWB64048	A/C	5BL_10864426	5BL	141.7	125.7
		D_GDRF1KQ01DK0HR_216	IWB19179	T/C	5DL_2218680	5DL	-	150.9
		GENE-2436_70	IWB32929	A/G	5DL_3476694	5DL	-	-
*AO-2*	Aldehyde oxidase 2	Ex_c12642_2562	IWB19755	T/C	2AL_6408908	2AL	-	-
		Ku_c11131_911	IWB38213	A/G	2AL_6408908	2AL	-	-
		Kukri_c9197_1341	IWB48277	T/C	2BL_8081481	2BL	93.2	99.2
		Ra_c16283_1844	IWB51213	A/C	2BL_8081481	2BL	-	-
		Kukri_c9197_1171	IWB48276	A/G	2BL_8081481	2BL	-	-
		Excalibur_rep_c111561_524	IWB30538	A/G	2BL_8003581	2BL	-	-
		Kukri_rep_c101425_563	IWB48641	T/C	2BL_8003581	2BL	-	-
		RAC875_c16283_179	IWB54261	A/C	2BL_8003581	2BL	-	-
		Excalibur_rep_c111561_524	IWB30538	A/G	2BL_8003581	2BL	-	-
		Kukri_rep_c101425_563	IWB48641	T/C	2BL_8003581	2BL	-	-
		RAC875_c16283_179	IWB54261	A/C	2BL_8003581	2BL	-	-
		RAC875_rep_c106900_118	IWB61642	T/C	2DL_9906604	2DL	-	-
*AO3*	Aldehyde oxidase 3	RAC875_c64451_465	IWB59875	T/C	7AL_4455104	7AL	180.3	-
		Kukri_c55360_910	IWB46506	T/C	7AL_4455104	7AL	-	-
		Kukri_c55360_1138	IWB46505	A/G	7AL_4455104	7AL	-	-
		Kukri_c5789_964	IWB46700	T/C	7AL_4455104	7AL	-	-
		Kukri_rep_c103039_607	IWB48835	T/C	7AL_4455104	7AL	-	-
		Excalibur_rep_c112889_341	IWB30603	A/G	7AL_4455104	7DL	-	184.2
		RAC875_c15047_109	IWB54046	T/C	7BL_6713884	7BL	-	-
		Excalibur_c2883_158	IWB24759	A/C	7BL_6641589	7BL	-	-
		Ku_c5789_663	IWB39661	A/G	7BL_6641589	7BL	-	136.3
		Kukri_rep_c110445_289	IWB49452	T/C	7BL_1920530	7BL	-	-
		Excalibur_rep_c104090_439	IWB30010	A/G	7DL_3396507	7BL	-	120.8
		wsnp_Ex_rep_c104090_88891443	IWB79099	A/G	7DL_3396507	7BL	-	121.2
		Ku_c5789_1180	IWB39660	A/C	7DL_3391726	7BL/7DL	155.7	120.8
		Kukri_c5789_1029	IWB46699	T/C	7DL_3391726	7AL/7DL	180.3	182.3
		RAC875_c89296_456	IWB60958	T/C	7DL_3391726	7DL	-	-
		Excalibur_rep_c101576_466	IWB29738	T/C	7DL_3391726	7DL	-	-

### Characterization of wheat AO3 gene sequences

The genomic sequence and structure of *AO-A3, AO-B3*, and *AO-D3* genes was investigated in the cv Chinese Spring in order to study the possible relationships between *AO3* genes and carotenoid accumulation in the wheat grain (Colasuonno et al., 2014). Relying on the Softberry calculation regarding the 7AL-4455104 contig, the *AO-A3* genomic sequence was 6,749 bp long with 42% GC content. The predicted gene sequence included an mRNA of 3,786 bp and a protein length of 1,262 amino acids. For *AO-B3*, the partial sequence included in the contig 7BL_6713884 was implemented by the complete EST sequence AK331622 (from NCBI database) and allowed to obtain a cDNA of 4,279 bp. The prediction of *AO-D3* gene, considering the 7DL_3391726 contig, presented a genomic sequence of 6,215 bp with an mRNA length of 4,038 bp and a protein 1,346 amino acids. A similar intron/exons structure was predicted between the wheat *AO3* genes composed by 10 exons and 9 introns sharing an identity of > 80% among homoeologues and 97% between *AO-B3* and *AO-D3*. The Fgenesh++ gene prediction pipeline highlighted a lack of similarity for the first and last exons between *AO-A3* and *AO-B3/AO-D3*. Only the last exon displayed high identity (100%) between *AO-B3* and *AO-D3* isoforms.

Furthermore, the wheat *AO3* cDNA alignment showed a smaller region (81 bp) in exon 6 for *AO-A3* than in exon 6 for *AO-B3* and *AO-D3*, and a corresponding longer region in the adjacent intron. This difference suggested an alternative splicing site and the sequencing analysis of cv. Chinese Spring cDNAs confirmed this length polymorphism among genomes.

**Figure 3:**
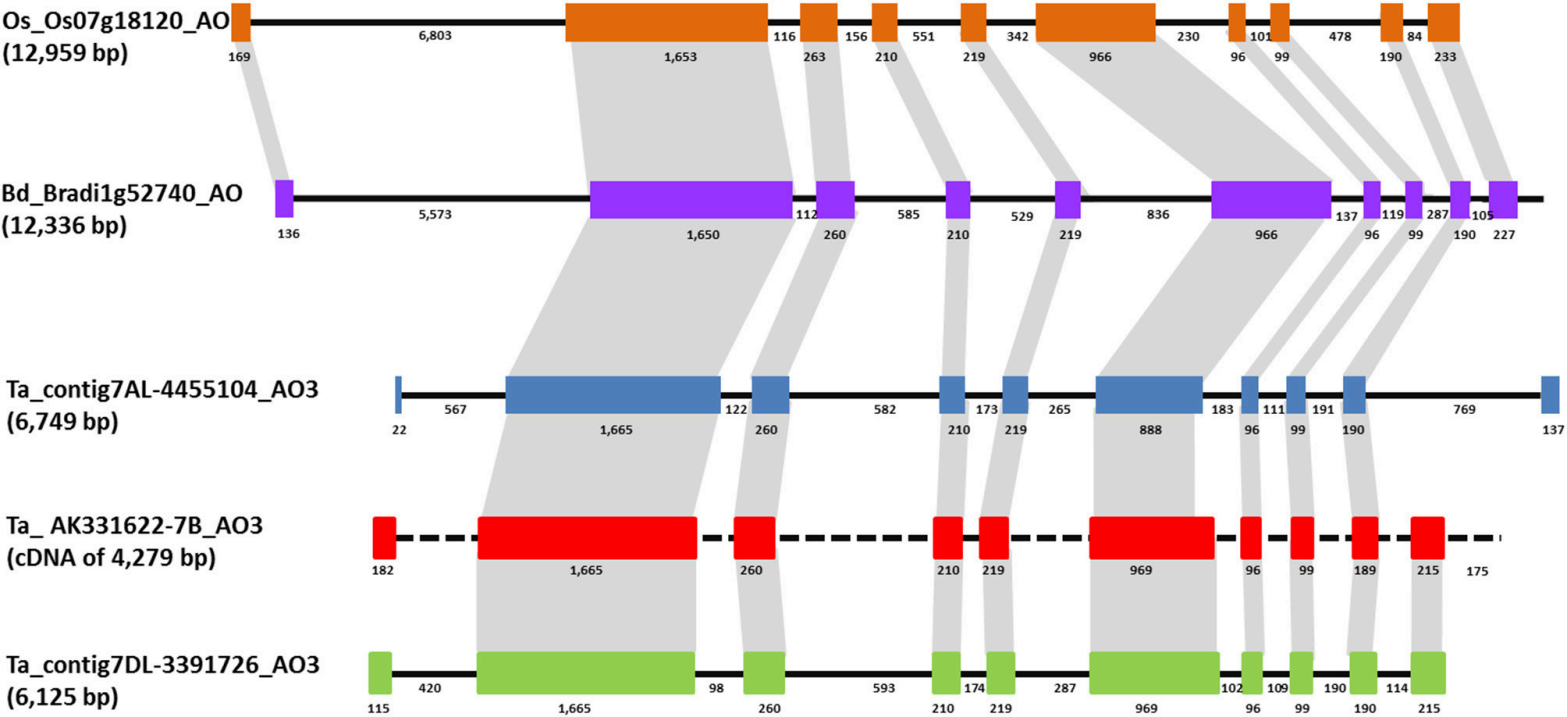
**Comparison of ***AO3*** gene structures in rice, ***Brachypodium***, and wheat is shown based on colored boxes highlighting conserved exons**. Intron and exon sizes are shown as well as the whole gene (in brackets the total length). Rice and *Brachypodium AO* share the same structure with ten exons of conserved sizes and nine introns. *Brachypodium* and wheat *AO3* show an high similarity in sequence and structure for eight exons. Black dashed line indicates the absence of intron sequence since only a cDNA sequence has been found for *AO-B3*.

BLAST analysis using Phytozome v.7 software (http://www.phytozome.net) with *B. distachyon* and *O. sativa* genomes allowed the comparison of wheat *AO3* genes with the orthologous genes located on chromosome 1 of *Brachypodium* (locus name: Bradi1g52740) and chromosome 7 of rice (locus name: Os07g18154). The *Brachypodium AO* consist in a sequence of 12,336 bp with a CDS of 4,053 bp, whereas in rice genome *AO* had a sequence of 12,959 bp with a CDS of 2,535 nucleotides. Comparison between wheat and *Brachypodiun* genomic AO sequences showed identities of about 84%, and an identity of 89% between the two CDS. A similar intron/exons structure was observed between *AO3-A1* from wheat and *AO* from *Brachypodium* composed by 10 exons and nine introns, except for the first and last exons (Figure 3). An identity of 79% was found aligning the *AO-A3* genomic sequence with the rice AO sequence.

In order to investigate the role of promoter region, a bioinformatics analysis was conducted in public database and a region of 1,600 bp (from contig7AL_4455104) upstream of the AO-A3 was identified. The analysis through Softberry showed clearly the presence of a promoter regions upstream the gene (data showed in Supplemental Material S2). Moreover, PlantCARE revealed the presence of 32 elements that are characteristic of promoters. These elements include the A-box, ARE, Box4, BoxI, Box-W1, CAAT-box, CAT-box, CATT-motif, CCAAT-box, CCGTCC-box, CGTCA-motif, CTAG-motif, G-box, GAG-motif, GARE-motif, GC-motif, GCN4_motif, GT1-motif, I-box, P-box, Skn-1_motif, Sp1, TATA-box, TCA-element, TCCACCT-motif, TCT-motif, TGACG-motif, TGG-motif, Wbox, WUN-motif, boxE, boxes, and circadian.

Further investigation through the Plant MITE database revealed the presence of two transposon regions. The first one was located at 336 bp and belonged to MITE family DTC, the other one at 901 bp position corresponded to MITE family DTT. The promoter region sequences in the cvs Ciccio and Svevo relieved absence of significant polymorphisms. According to this, the differences on the expression levels among cultivars could be due to a different regulatory mechanisms in action inducing different genes.

### Expression profile of AO genes in wheat

**Figure 4:**
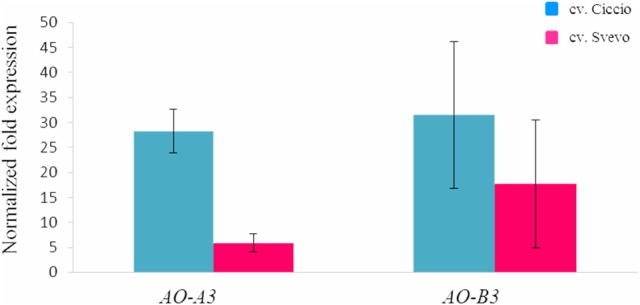
**Comparison of the expression levels of ***AO-A3*** and ***AO-B3*** genes in kernel tissue of cv. Svevo and cv. Ciccio by qRT-PCR**. The y ax shows the normalized fold expression. The error bars indicate the ± SE of the mean.

The possible relationships between AO genes and carotenoid accumulation was investigated in the wheat grain. The *AO-A3* and *AO-B3* gene expression levels were determined in two durum wheat cultivars, Ciccio and Svevo, characterized by a low and high YPC, respectively. Total RNA was extracted from kernels, and quantitative real-time PCR was conducted with specific primers in order to analyze individually the two homeologous *AO3* genes. High expression levels were observed for *AO-A3* and *AO-B3* during seed maturation stage in Ciccio, while low amounts were detected in Svevo (Figure 4). Significant different expression values (*P* < 0.001, *t*-test) were observed between the two cultivars only for *AO-A3*. The data suggested that the *AO-A3* allele present in Ciccio (cultivar characterized by a low content of YPC) was more active into carotenoid degradation, while the Svevo (high YPC) allele was not fully involved in the carotenoid catabolism.

**Figure 5:**
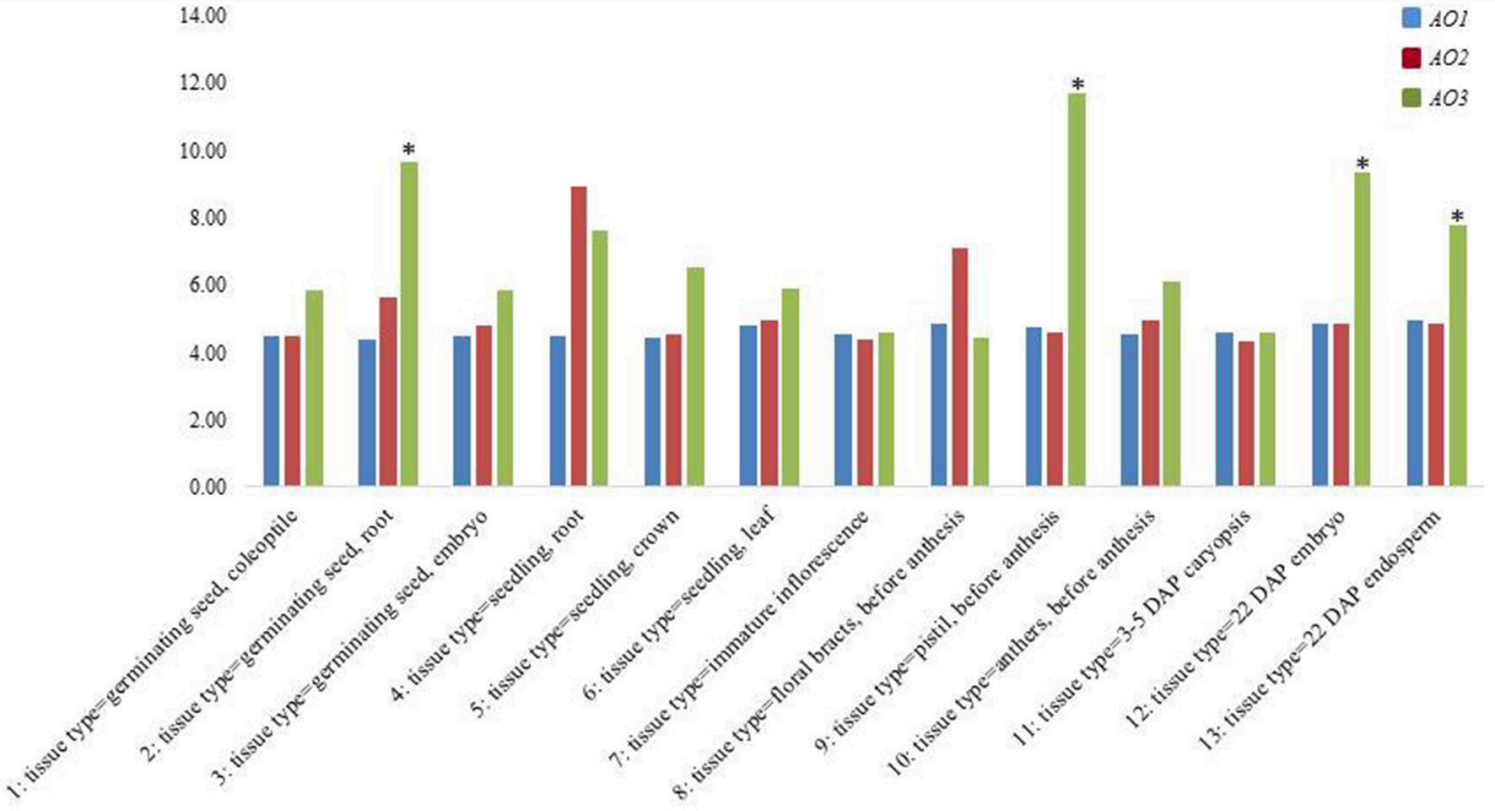
**Expression analysis from PLEXdb database of all wheat ***AO*** genes in a wide range of tissues and developmental stages in wheat (1–13)**. The asterisk signals indicate, respectively the values higher or lower than the mean values ± 2 *SD*.

In order confirm our data and to understand the expression trend of wheat *AO* genes, a check on data available in the PLEXdb database was carried out considering the cDNAs included in the largest contigs (2AL_6408908, 5BL_10864426, and 7AL-4455104) chosen as representative of each wheat homoeologous group (Figure 5). The Wheat 61k GeneChip showed different expression pattern of *AO* genes during developmental stages underlining how *AO2* and *AO1* resulted constantly expressed in all stages with maximum levels of 8.93 RMA normalization for *AO2* in seedling root (phase 4). Instead, *AO3* showed significantly high expression levels (values higher than the mean values ± 2 SD) especially in last phases of embryo and endosperm kernel formation (phase 9, 12, and 13) indicating a major role in the last developmental stage of wheat seeds.

### Development of a functional AO3 marker

**Figure 6:**
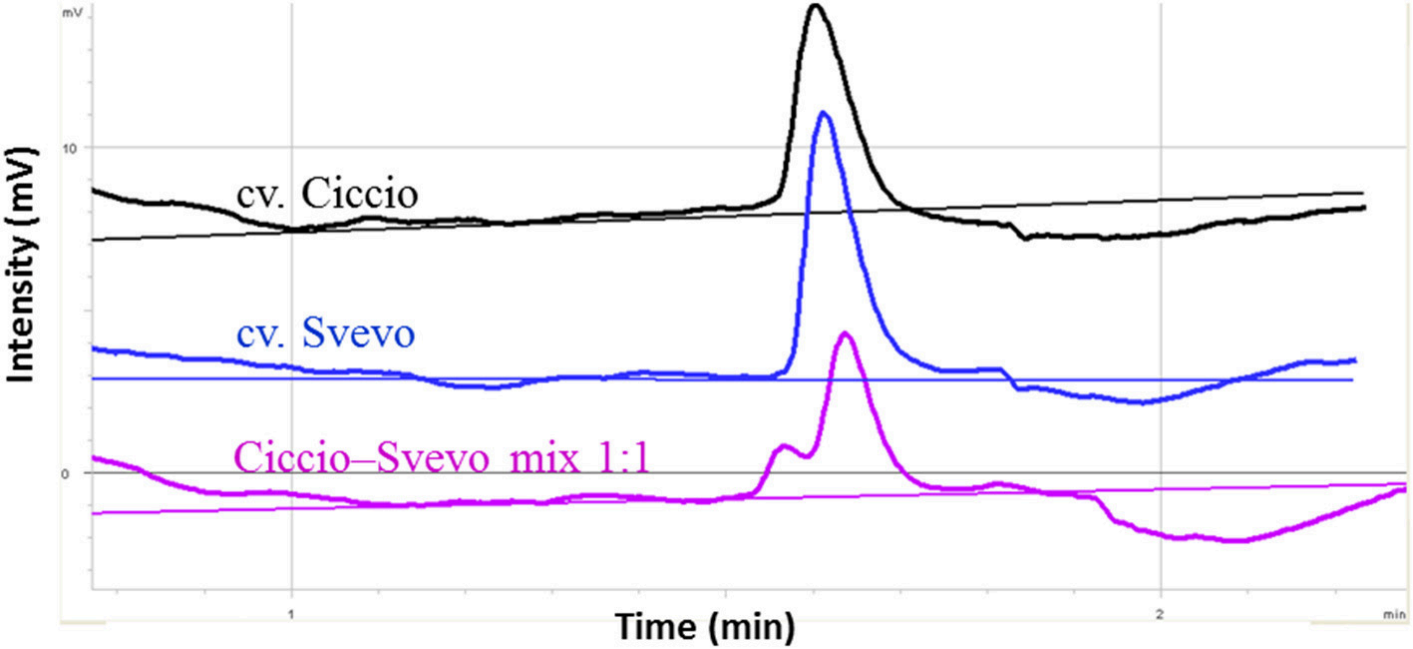
**Optimization of DHPLC analysis for SNP detection between cvs. Ciccio and Svevo at locus IWB59875**. The chromatograms correspond to the elution profiles of homoduplex molecules (cv Ciccio in black, cv Svevo in dark blue), and of homoduplexes plus heteroduplexes derived from 1:1 mixed DNA (pink line).

A considerable emphasis has been placed on the *AO-A3* gene for the development of molecular markers to be used for MAS in wheat breeding. Due to the availability of SNP data corresponding to this isoform, the IWB59875 marker mapped on chromosome 7A and resulted associated to carotenoid content (Colasuonno et al., 2014) was chosen for setting up a SNP-based method suitable for wheat breeding. Before proceeding into the analysis of DNA fragments, PCR primers specific for *AO-A3* gene were designed respecting the DHPLC conditions, such as the distance of 50 bp from the SNP site and a fragment size ≥ 200 and ≤ 800 bp. After that, the DHPLC technique was optimized for the run conditions on each amplicon, considering the optimal temperature (55°C). According to the basic principle of the DHPLC technique, if the sample contained the DNA from a homozygous genotype, the chromatogram showed a single peak derived from only homoduplex molecules. The homoduplex originated from Ciccio was practically undistinguishable from that of Svevo, having the same retention time. On the contrary, when the sample was a mix of two cultivars, and a SNP was present, the chromatogram exhibited two peaks of similar area, one corresponding to the coeluting homoduplex molecules (imputable to both Ciccio or Svevo with themselves), the other to the early elution of the heteroduplex molecules (due to Ciccio DNA combined with that of Svevo). As shown in Figure 6, the DHPLC allowed detecting two chromatographic peaks corresponding to heteroduplex and homoduplex molecules derived by the presence of the T/C substitution (detected by the IWB59875 marker) in the two durum varieties. The regression analysis conducted between the AO-A3 gene marker and YPC confirmed the association with the trait with an high LOD scores in all the four environment considered and in the mean across them. The phenotypic variation explained by the gene marker (R^2^) ranged from 18.0 to 41.0% and the effect due to the Ciccio alleles was reported for all the environment analyzed in Table 2.

**Table 2:** **Regression analysis between the SNP marker IWB59875 for the gene abscisic aldehyde oxydase 3 (AO-A3) and yellow pigment content evaluated in the Svevo × Ciccio RIL population grown in four environments**.

**Environment**	**Effect**	**−log10(P)**	***R*^2^**
Valenzano 2006	−0.58	8.1	0.30
Foggia 2006	−0.45	4.6	0.18
Valenzano 2007	−0.72	11.9	0.41
Foggia 2007	−0.48	4.4	0.18
**Mean across environments**	−**0.56**	**7.7**	**0.29**

*R^2^, Phenotypic variation explained by the QTL*.

*The mean values across enviroments are reported in bold*.

## Discussion

High levels of carotenoid pigments in wheat kernels have important positive implications for human health since they are antioxidant compounds and precursor of vitamin A. Knowing the role of the main carotenoid genes in wheat and the specific alleles present in each cultivar can allow to develop superior cultivars through marker-assisted breeding programmes.

Several studies reported many QTL for carotenoid content spread all over the wheat genome (reviewed by Colasuonno et al., 2017). Among them, two different QTL were mapped on the long arm of chromosome group 7, co-localized with phytoene synthase 1 (*Psy1*) and aldehyde oxidase 3 (*AO3*) genes, respectively (Zhang and Dubcovsky, 2008; Blanco et al., 2011; Colasuonno et al., 2014, 2017). While the *Psy1* involvement in YPC has been deeply studied, the role of *AO3* gene in the carotenoid accumulation needs to be elucidated. AO isoforms are key enzymes for ABA biosynthesis (Fang et al., 2008; Zdunek-Zastocka, 2010). Plant AO family is composed by proteins with high similarity in sequences, but different subunit composition and substrate preferences. AO isoforms have been largely characterized in *Arabidopsis* and resulted composed by 4 isoforms (Seo et al., 2000a). While significant information exists on *AO* families in *Arabidopsis*, lettuce, tomato, peas, Brassica, rice and maize, a complete picture of this family is missing in wheat (Zdunek-Zastocka, 2010).

Overall, 34 SNPs within AO wheat sequences were identified, making significant advancement on the localization of AO genes on wheat chromosome groups. Indeed, the recent availability of the high-resolution consensus map of durum (Maccaferri et al., 2015) and bread wheat maps (Wang et al., 2014) allowed to determine the precise map position. The *AO3* mapping was consistent with results reported by Colasuonno et al. (2014, 2017), who identified the chromosomal locations based on survey sequence from the International Wheat Genome Sequencing Consortium (http://www.wheatgenome.org/). Instead, the map positions of *AO2* and *AO1* genes were reported for the first time: *AO2* localized on chromosome group 5 and the *AO1* on chromosome group 2.

The present study considered the AO gene expression in wheat. AO isoforms were found expressed in root tissues under osmotic stress (Gallè et al., 2013). The *AO2* and *AO1* genes resulted expressed in all stages with maximum levels for *AO2* in seedling root. While, the *AO3* showed elevated expression levels during kernel maturation with a possible involvement in carotenoids pathway since they are accumulate at last developmental stage.

The first connection between carotenoid content and the *AO3* genes emerged form a genetic study on QTL in a RIL population derived crossing the durum wheat cvs Ciccio and Svevo (Colasuonno et al., 2014). Previously, other studies (Zhang and Dubcovsky, 2008; Blanco et al., 2011) indicated the presence of this second QTL for carotenoids, using different genetic materials. Then again, Colasuonno et al. (2017) showed that *AO-A3* gene was significant associated to carotenoid variation using GWAS and the “candidate gene” approaches.

Based on the evidence presented, we have envisaged that mutations in *AO3* induce activity-loss in carotenoid catabolism from violaxanthin/neoxanthin to (ABA) and allows an accumulation of carotenoid compounds. The transcriptional level of the *AO-A3* and *AO-B3* genes in the cvs Ciccio and Svevo was in accordance with this hypothesis, demonstrating how *AO-A3* gene resulted in significant low expression levels in Svevo, cultivar with high content of carotenoids. This could be explained by the presence of an *AO-A3* allele in the cv Svevo not fully activated for carotenoid catabolism and ABA biosynthesis. The *AO3* analysis need to be further examined to evaluate how the ABA concentration differs in relation to allele functionality. Indeed, in *Arabidopsis* and maize (Wurtzel et al., 2012; Gonzalez et al., 2013) the carotenoid degradation is important in determining total carotenoid accumulation.

In addition, the regression analysis conducted in the RIL population confirmed the association between the AO-A3 gene marker and YPC. Besides, the development of a molecular gene marker for *AO-A3* through the most sensible and cost-effective technique (such as the DHPLC) demonstrated for the first time its applicability on marker assisted selection programmes (MAS). Giancaspro et al. (2016) used the same technology as tool for SNP detection based on genotyping arrays in food science.

The present work characterizes suitable AO3 genes providing a new insight into the regulation of carotenoid accumulation. Although the gene expression analysis revealed differences between the two cultivars, no polymorphisms were observed in the promoter regions, suggesting the presence of complex gene regulation mechanisms. The factors influencing pigment content are complex (Howitt and Pogson, 2006; Lachman et al., 2017). Further investigations needed in order to understand the carotenoid pigment regulation system in wheat.

## Author contributions

PC, AB, and AG designed the research; IM and ML performed the research. PC and RS wrote the manuscript. All authors read and approved the final manuscript.

### Conflict of interest statement

The authors declare that the research was conducted in the absence of any commercial or financial relationships that could be construed as a potential conflict of interest.
