# Supplementary material for: Characterization of Aldehyde Oxidase (AO) Genes Involved in the Accumulation of Carotenoid Pigments in Wheat Grain
Source: Front Plant Sci. 2017 May 24;8:863. doi: 10.3389/fpls.2017.00863 (PMC5443152; doi:10.3389/fpls.2017.00863)
Supplement: Supplementary file 1 [file Table1.DOCX]

**Table S1.** Primer pairs specific for the AO genes of wheat

| **Gene name** | **Primer orientation** | **Primer 5'-3' sequence** | **Product length (pb)** | **Melt peak (°C)** |
| --- | --- | --- | --- | --- |
| *AO-A3* | Fw | TGGAAGGAACATCACATGCTG | 196 | 81 |
|  | Rv | TGATCACCACTGGAAACTGTG |  |  |
| *AO-B3* | Fw | ATACAGAGTCAGTGTGGCCG | 174 | 80.5 |
|  | Rv | TGATCATCGCTGGAAATTGTT |  |  |
