# Supplementary material for: Characterization of Aldehyde Oxidase (AO) Genes Involved in the Accumulation of Carotenoid Pigments in Wheat Grain
Source: Front Plant Sci. 2017 May 24;8:863. doi: 10.3389/fpls.2017.00863 (PMC5443152; doi:10.3389/fpls.2017.00863)
Supplement: Supplementary file 2 [file Table2.DOCX]

**S2**.Upstream region of AO-A3 gene. The MITE family DTT has been reported in orange letters and the MITE family DTC in pink.

> Chinese Spring_contig7AL_4455104 partial sequence

ATGCAAAAGTTATAGCCGTTTTACTTTTTCATGACACTTTTTTGCAAAACATGTTCAAATTTAAGTTTTTTTATTTTCCTAACTAGTAGATGTAGTAATATAACTACATCTCGAAGGATTTTATTTTTTGAAGTTTTTATCATTTTCTTTTGTTTTTTTCAAAACTGAAATGGCGATACACCCGGGGGGTAGAGTTTGAGAAATGGGCCCTTTAGTCCTGGTTTGAGACACGAACTGGGACAAAAGGGCATCGCACCCTTTAGTCCCGATTCGTGTGTCAAACCGGGACTAAAGGGCCCATTTGAACCAGGACTAATGCCTTTAGCCGCTTGAACCGGGACTAATGCTATATTAGTCCCAGTTCGTAATGCAATCGGAACTAATGTGTATATTGCGCTGTGACCAAAGCCCTGTTTTCTACTAGTGGCTCCACCTGGATAATATATTCCCGGTCTAGCGCAAGTCAATGTCTTCCACCCCACCAATTTCTTAGCTCAATGAGCCTGCTAGAACTTATGGCAACCGCACCACTAAGATTGAGCTCCTCCCCGTTGGACCTGCTCTTGGTAGCGCCATTGGTAACGGCCTCTCCTTCTCATAGGCTCAAAACCCTCAAAAGGTCAGTACATCCACAATGTACATTGAGCCAATGTAAGTATGCTCTACATTTTAGAACAAGACCTAATGAGTTACTACCTACGTTTCAAAATAAGTGAAGTTCTACTTTGTCCTTGGTCAAACTTGTTTAAGCTTGACTAACCCTATAGAAAAATATGTCAAGATCTACAACACAAATACACTAGTGCAAAAATAATCTCATGAGACTAATTTGATGTTCTAGATGTTAATATGTTTTTCTATAGACTTTGTTAAATTTACACAAATTTGACTTAGGAAAAATTTAGAACTTTAATTCTTTTGAAACGGAAGTAGCATCTCTTAAGCTAAGGATCCAAAGTGACAATTTAACCACATAAGTGGTGGGTAGCATTTAACATGATAGCATTTGGATCAACAAAGATGTAGGGAAAGCTATTCAAAATTCGGCTTGATTTTGTATTATTTTTTTGTGGATTACACCAGCCTGATGAAATAGAGTTTGTGAAGATCACGAATCCCTGGTTTAGACACAAGTGCTCTCTCTATTAATTAATATAAAAGCCTTTAGATCACTATCACTAAATAGTGCTCTAAACACTCTTATATTTTTTACGGAGAAAGTATCATTACTACAAACTCCGCGGATGGTGATGCAATACTGCAAAAGTTTATGAAAAGAATAAAATGCACGGAATGCGTAAATTTGTTAGTGATGTTACTTTAAAAACAGACCCATCAACCATATCAATATTTGACAAAATCACAGGAATATTGCAAAAGAGCCCCTCATACCTCTGAGAGTAAAAACGGTACCCAAATTTCCCAAACCGGCCCTCAAATCCCTAGTTCTGCGTTGCCGCCGCACGTCTTCCCCAAATCCGGCGTCATCTCCGTCGAGCCACCGTGTCGTCCCTTTCCGTCCCCCGTCCCGTCCCCGCTCCCCTCGCTCTCCAATCTCTTACCATTTCTCTGCCTCCGCCCGCCCCCTCTGACCCAACCCATG
